# Supplementary material for: DNA polymerases in precise and predictable CRISPR/Cas9-mediated chromosomal rearrangements
Source: BMC Biol. 2023 Dec 8;21:288. doi: 10.1186/s12915-023-01784-y (PMC10709867; doi:10.1186/s12915-023-01784-y)
Supplement: Supplementary file 1 — Additional file 1: Fig. S1. DNA-fragment editing by Cas9 with dual sgRNAs. Fig. S2. HPRT1 and DCK reporter assay systems for Cas9-induced large resections. Fig. S3. Quantitative RT-PCR at two time points in HEC-1-B cells. Fig. S4. Additional examples of large resections by DNA sequencing during DNA-fragment editing. Fig. S5. High-throughput NGS of junctional sequences of DNA-fragment editing. Fig. S6. Significant decreases in the frequency of small deletions. Fig. S7 Significant increases in the frequency of precise ligations. Fig. S8. Quantitative RT-PCR at two time points in HEK293T cells. Fig. S9. Polλ enhances editing outcomes of 1bp deletions and suppresses the generation of >1bp deletions in HEK293T cells. Fig. S10. Biased deletion of nucleotides at junctional sites of chromosomal rearrangements confirms the staggered or cohesive Cas9 cleavages. Fig. S11. Fill-in of cohesive Cas9 DSB ends by Polλ in HEK293T cells. Fig. S12. Polδ, Polκ, and Polθ are not engaged in the fill-in of cohesive Cas9 cleavage ends. Fig. S13. PCR modelling-based analysis by the pcrEfficiency software. [file 12915_2023_1784_MOESM1_ESM.docx]

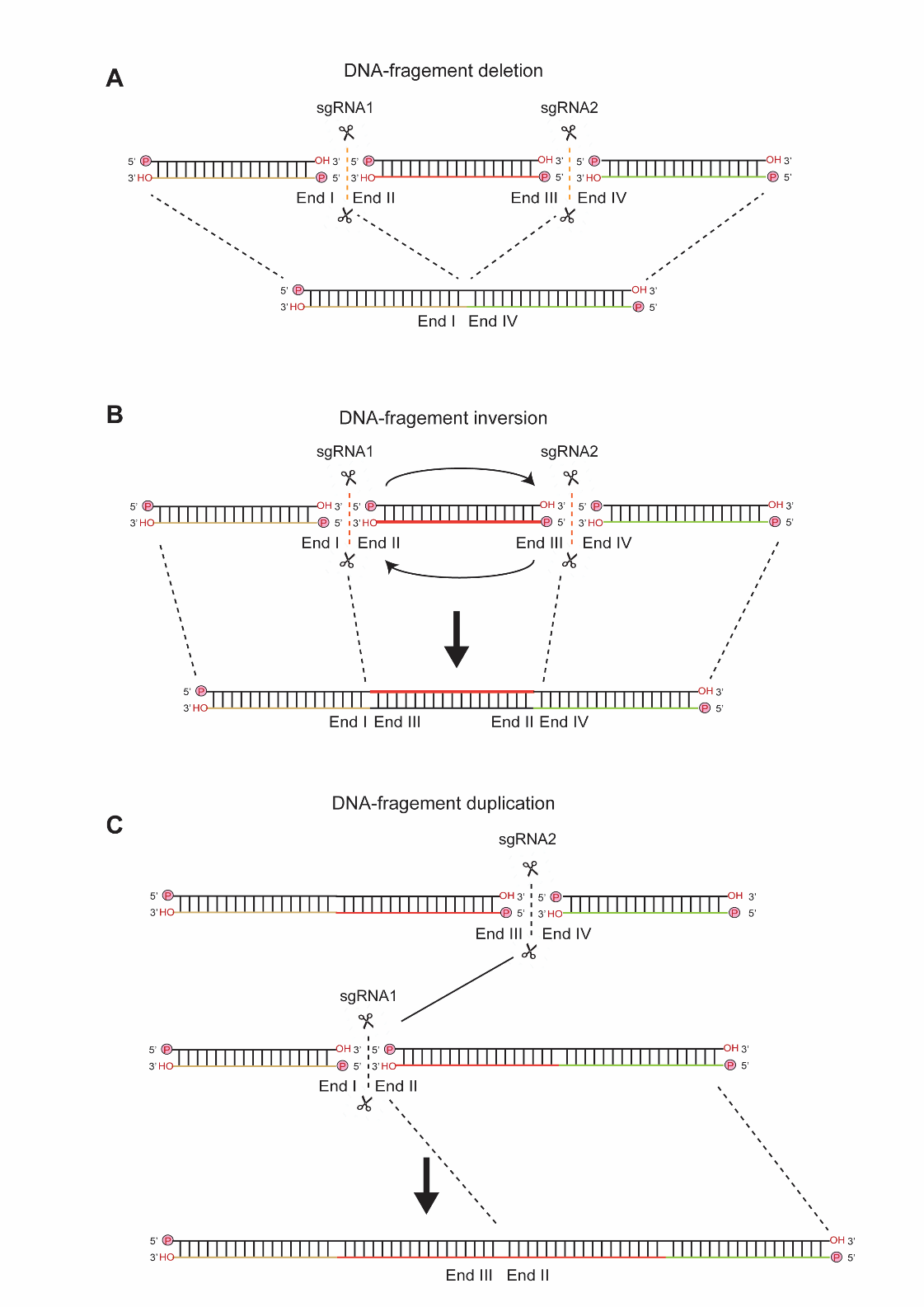
**Fig. S1** DNA-fragment editing by Cas9 with dual sgRNAs results in chromosomal rearrangements including DNA-fragment deletion, inversion, and duplication. Cleavages of Cas9 programed with dual sgRNAs result in two cuts with four double-stranded break (DSB) ends: I, II, III, and IV. (**A**) Ligation of DSB ends I and IV leads to DNA-fragment deletion. (**B**) Ligation of DSB ends I and III as well as ends II and IV leads to DNA-fragment inversion. (**C**) Transallelic ligation of DSB ends III with II leads to DNA-fragment duplication.


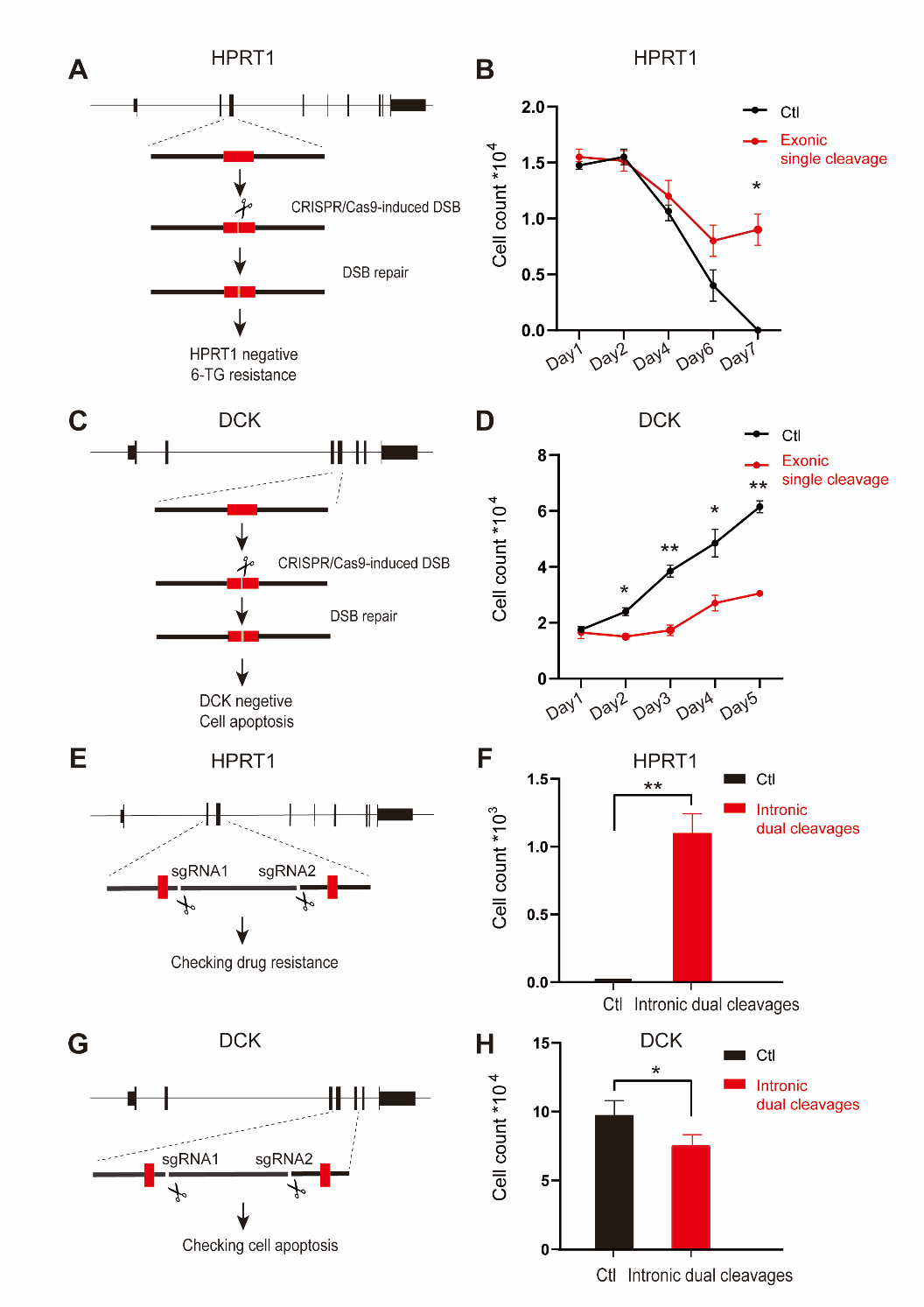


**Fig. S2** *HPRT1* and *DCK* reporter assay systems for Cas9-induced large resections. Schematics (**A**, **C**) and cell growth (**B**, **D**) of Cas9 with single sgRNAs targeting exonic sequences of *HPRT1* (with 6-TG) and *DCK*. Schematics (**E**, **G**) and cell growth (**F**, **H**) of Cas9 with dual sgRNAs targeting exon-proximal intronic sequences of *HPRT1* (with 6-TG) and *DCK* (see Additional file 5: Table S3, n = 2 replicates, mean ± SEM). Normal splicing of the intron with no large resection will not perturb *HPRT1* or *DCK* gene expression. Cas9-induced large resections into the flanking exonic sequences will disrupt normal splicing and perturb *HPRT1* or *DCK* gene function.


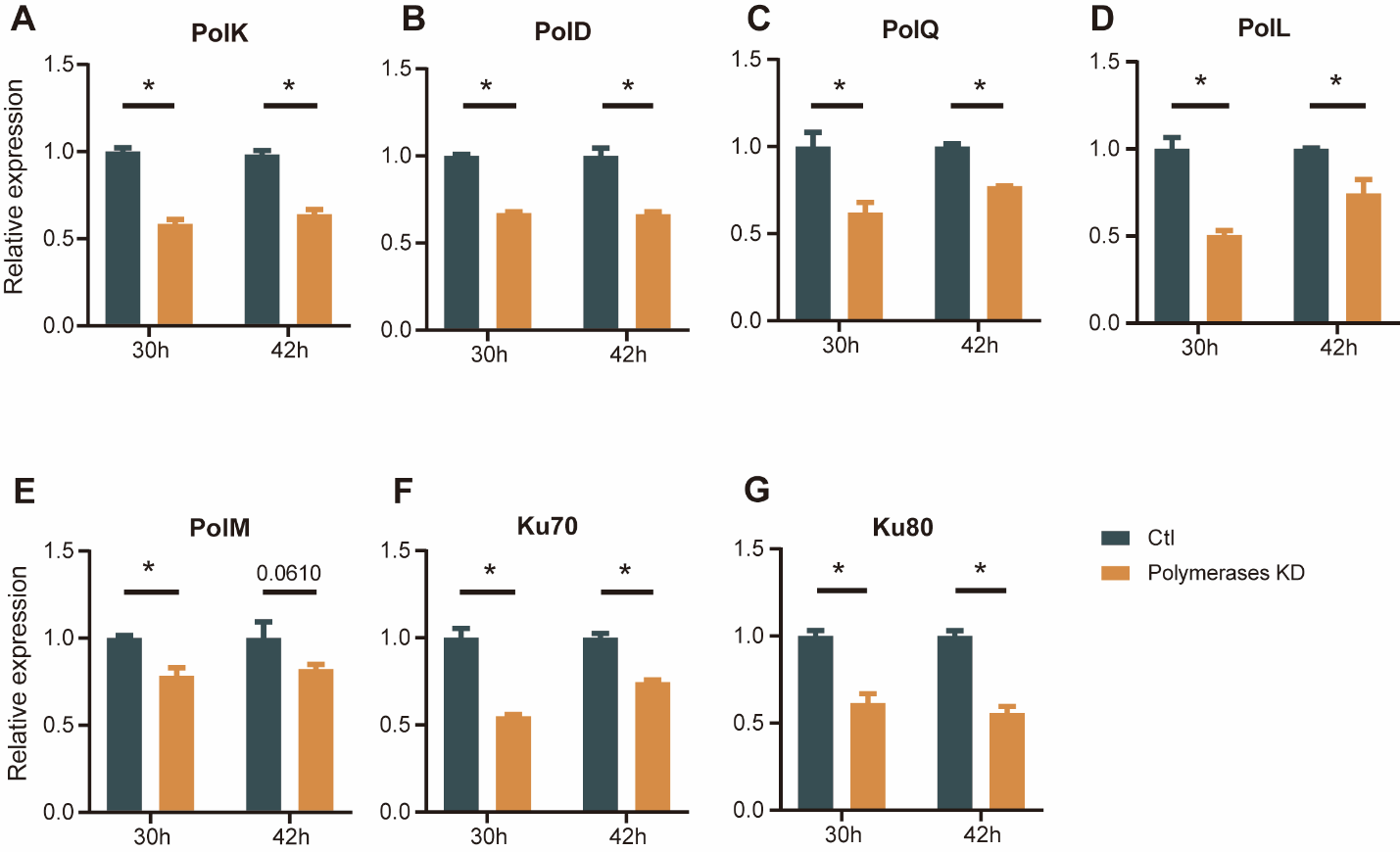


**Fig. S3** Quantitative RT-PCR at two time points upon knockdown (KD) of polymerases and Ku70/80 in HEC-1-B cells(see Additional file 5: Table S3, n = 2 replicates, mean ± SEM).


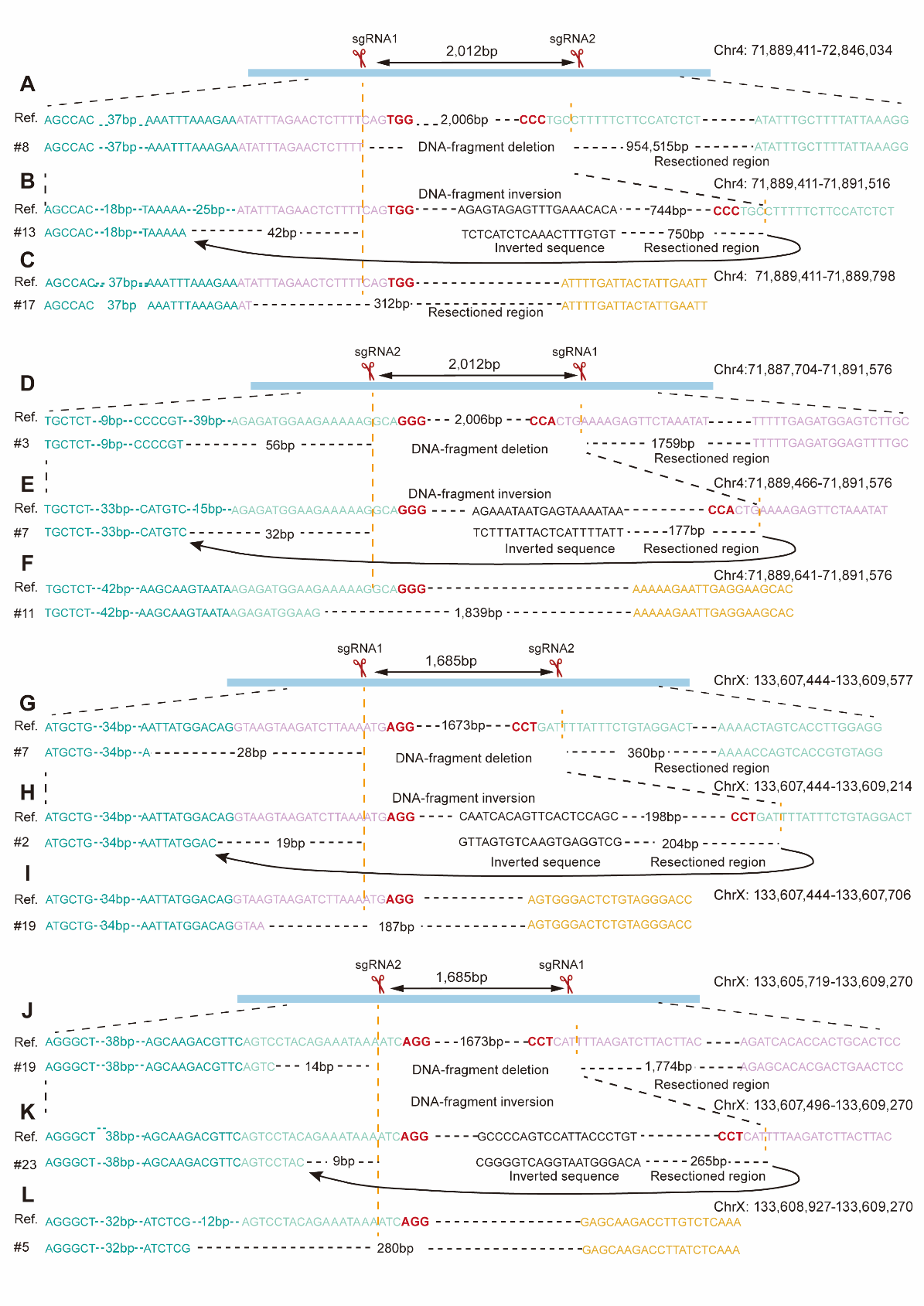


**Fig. S4** Additional examples of large resections by DNA sequencing during DNA-fragment deletion (**A**, **D**, **G**, **J**) and inversion (**B**, **E**, **H**, **K**) as well as at the upstream cleavage junction (**C**, **F**, **I**, **L**) programmed by Cas9 with dual sgRNAs at *DCK* (Chr4) and *HPRT1* (ChrX) loci.


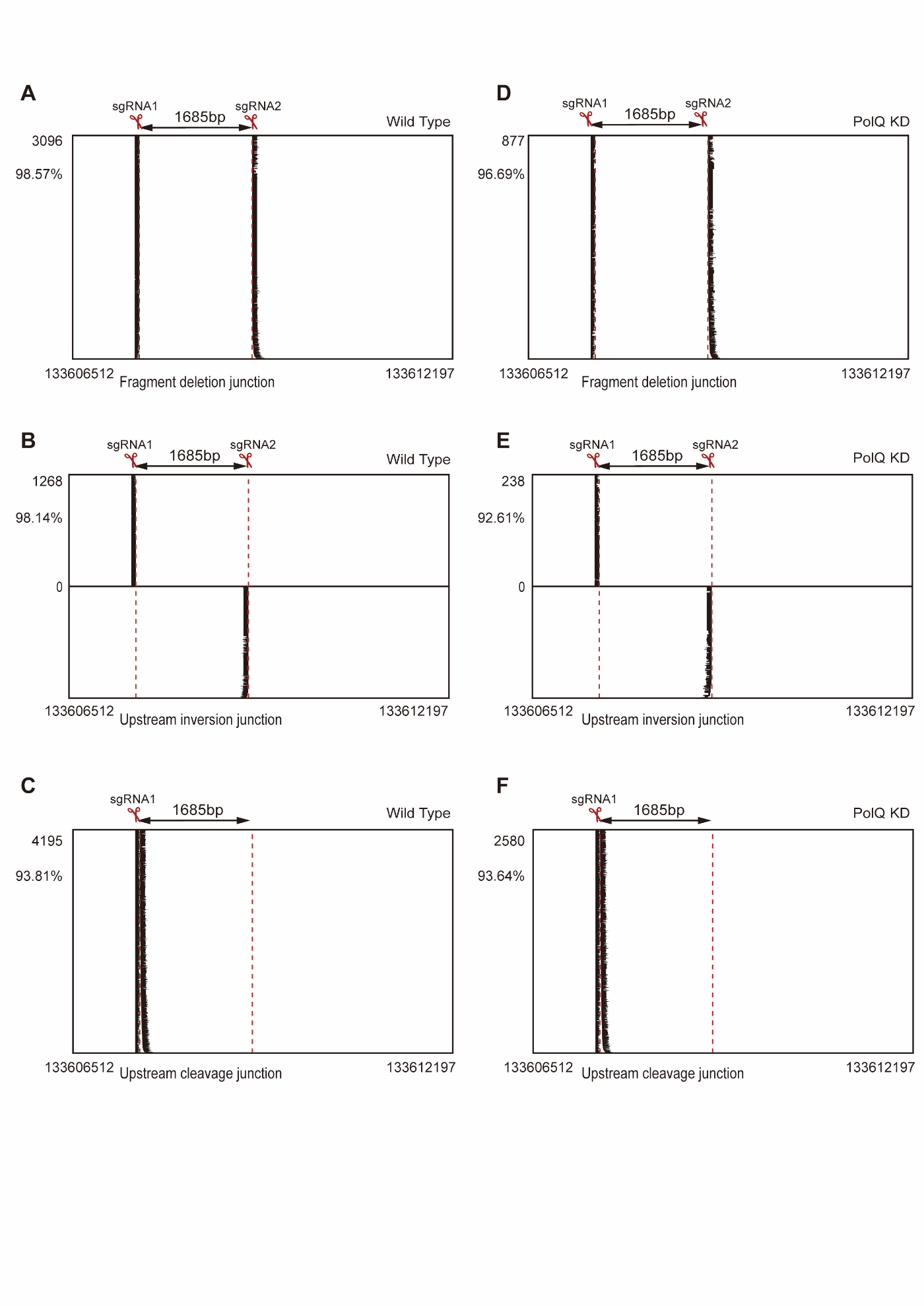


**Fig. S5** High-throughput NGS of junctional sequences of DNA-fragment deletions (**A**, **D**) and inversions (**B**, **E**) as well as of single cleavage junctions of Cas9 with sgRNA1 (**C**, **F**) upon *PolQ* knockdown. Note the more pronounced effects on chromosomal rearrangement junctions of DNA-fragment deletions and inversions (**A**, **B**, **D**, **E**) than on single cleavage junctions (**C**, **F**).


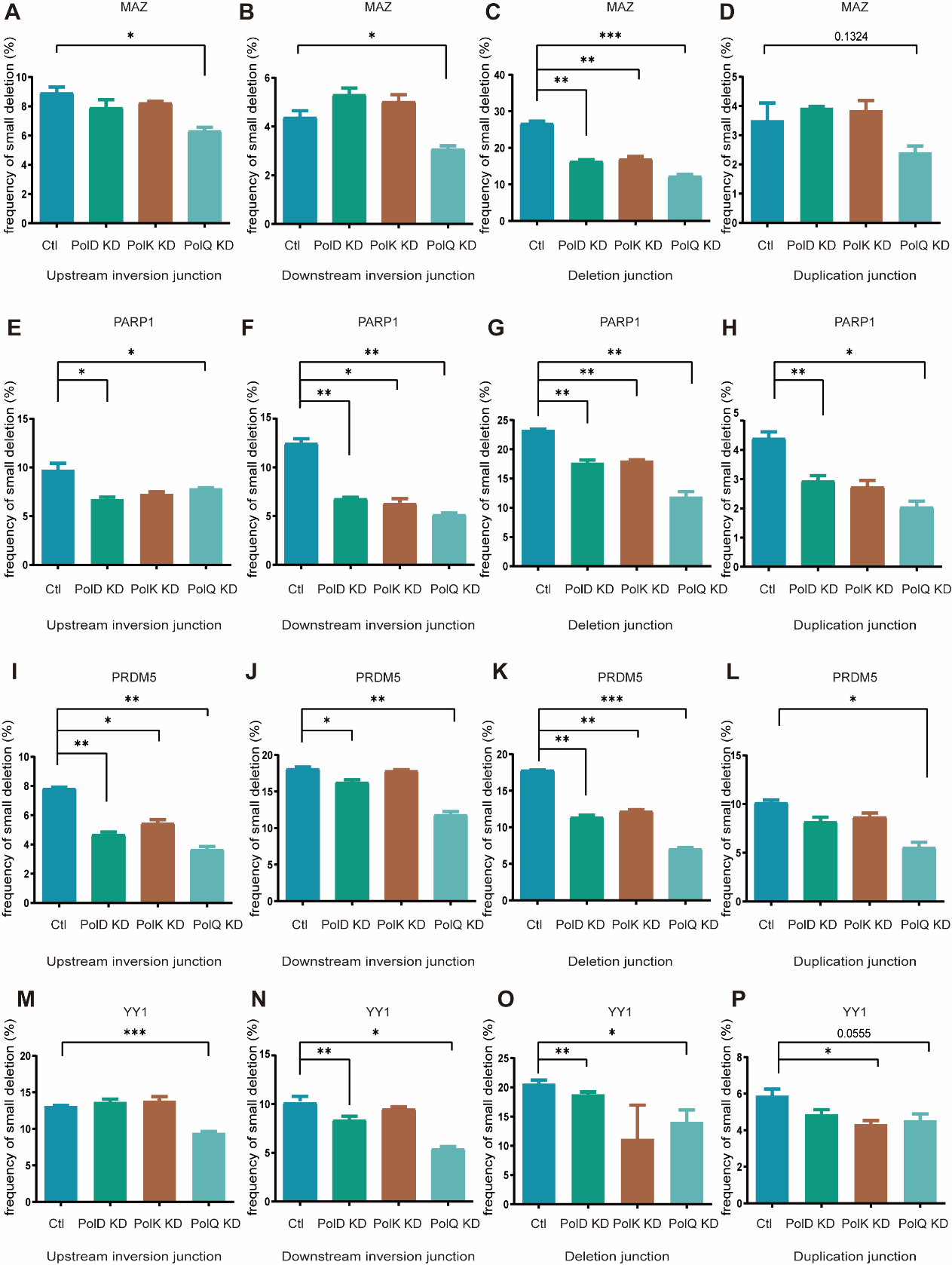


**Fig. S6** Significant decreases in the frequency of small deletions at upstream (**A**, **E**, **I**, **M**) and downstream (**B**, **F**, **J**, **N**) junctions of DNA-fragment inversion as well as at junctions of DNA-fragment deletion (**C**, **G**, **K**, **O**) and duplication (**D**, **H**, **L**, **P**) at the *MAZ* (**A**-**D**), *PARP1* (**E**-**H**), *PRDM5* (**I**-**L**), and *YY1* (**M**-**P**) loci upon *PolQ* knockdown (see Additional file 5: Table S3, n = 3 replicates, mean ± SEM).


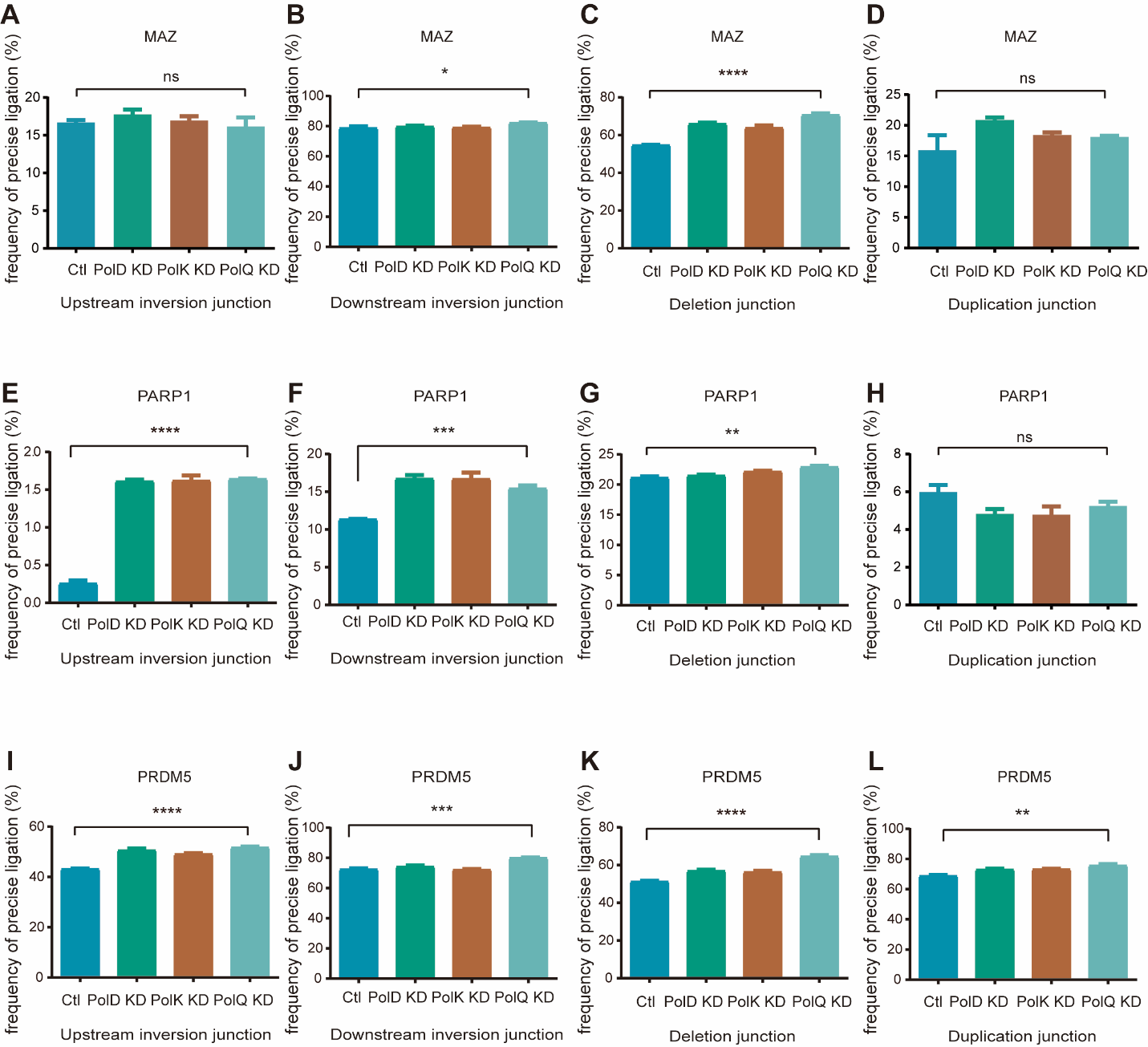


**Fig. S7** Significant increases in the frequency of precise ligations at upstream (**A**, **E**, **I**) and downstream (**B**, **F**, **J**) junctions of DNA-fragment inversion as well as at junctions of DNA-fragment deletion (**C**, **G**, **K**) and duplication (**D**, **H**, **L**) at the *MAZ* (**A**-**D**), *PARP1* (**E**-**H**), and *PRDM5* (**I**-**L**) loci upon *PolQ* knockdown (see Additional file 5: Table S3, n = 3 replicates, mean ± SEM).


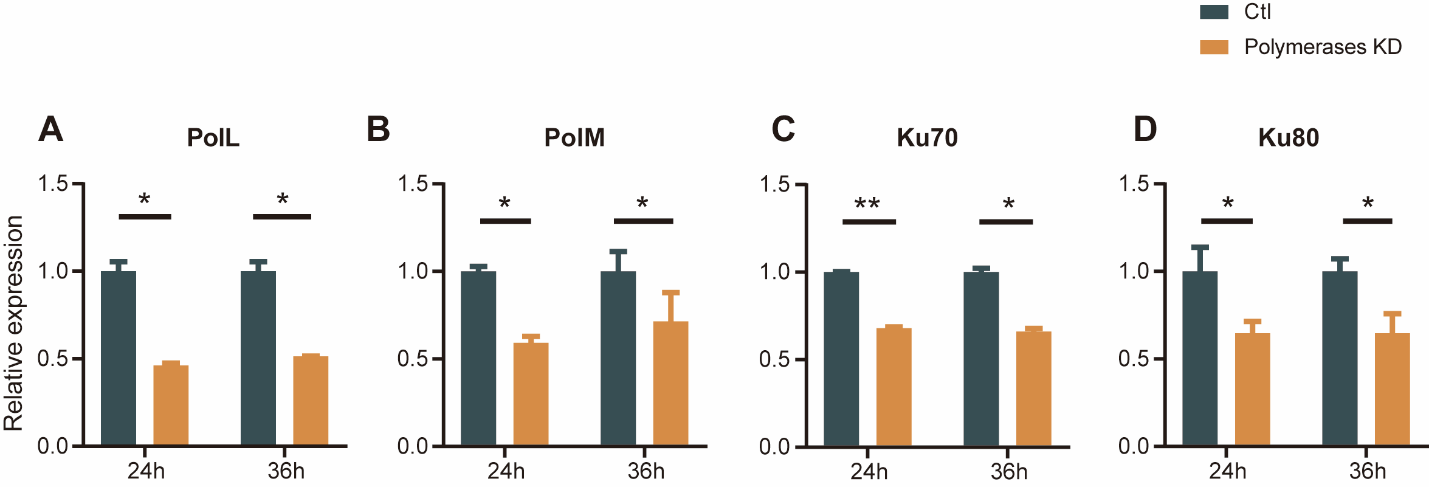


**Fig. S8** Quantitative RT-PCR at two time points upon knockdown of polymerases in HEK293T cells (see Additional file 5: Table S3, n = 2 replicates, mean ± SEM).


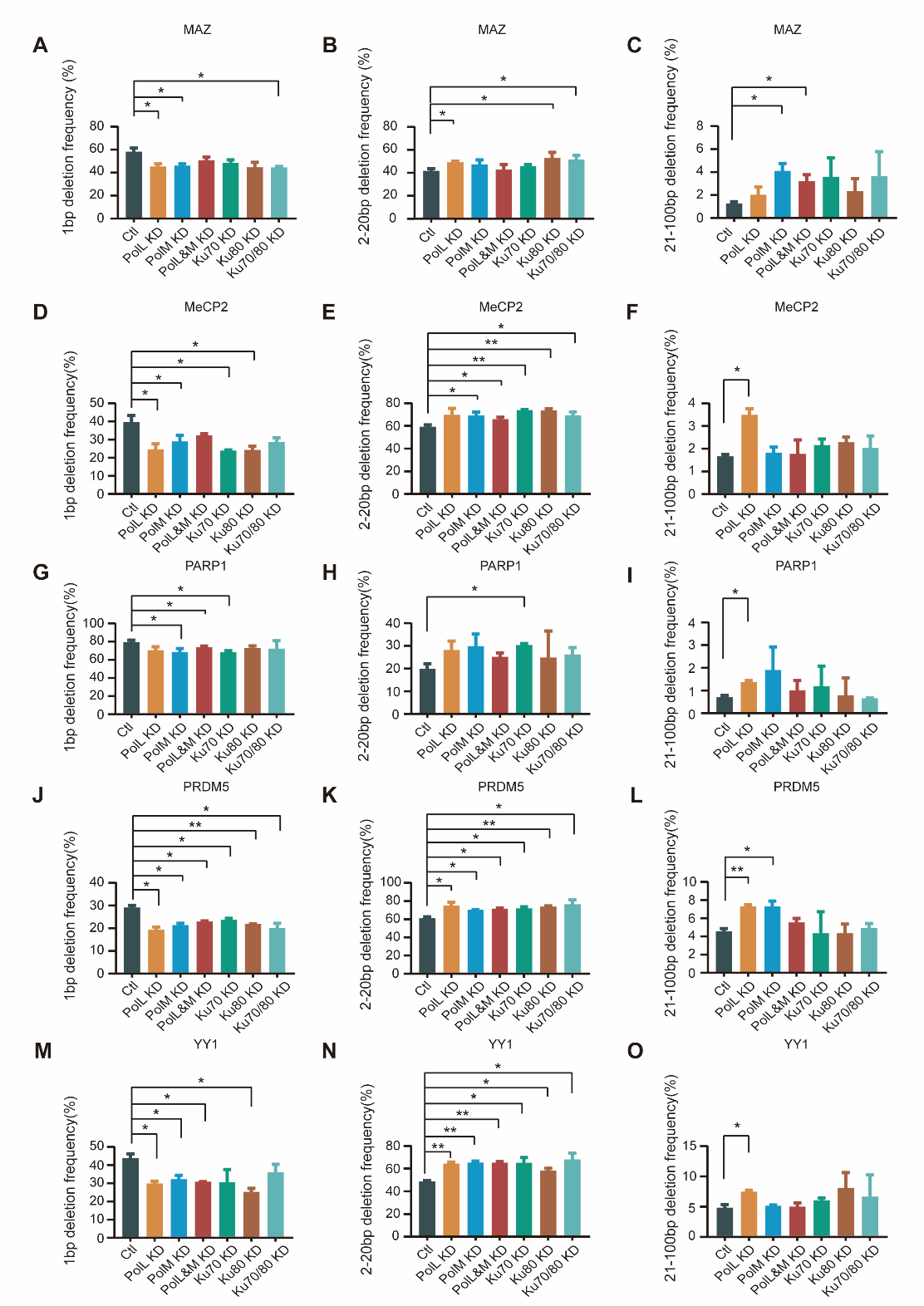


**Fig. S9** Polλ enhances editing outcomes of 1bp deletions and suppresses the generation of >1bp deletions in HEK293T cells. There is a significant decrease of frequencies of 1bp deletions (**A**, **D**, **G**, **J**, **M**) but an increase of frequencies of 2-20bp (**B**, **E**, **H**, **K**, **N**) and 21-100bp (**C**, **F**, **I**, **L**, **O**) deletions upon *PolL* knockdown at the *MAZ* (**A**-**C**), *MeCP2* (**D**-**F**), *PARP1* (**G**-**I**), *PRDM5* (**J**-**L**), and *YY1* (**M**-**O**) loci (see Additional file 5: Table S3, n = 2 replicates, mean ± SEM). As positive controls, the trends of small deletions upon Ku70/80-knockdown are similar as those of Polλ-knockdown.


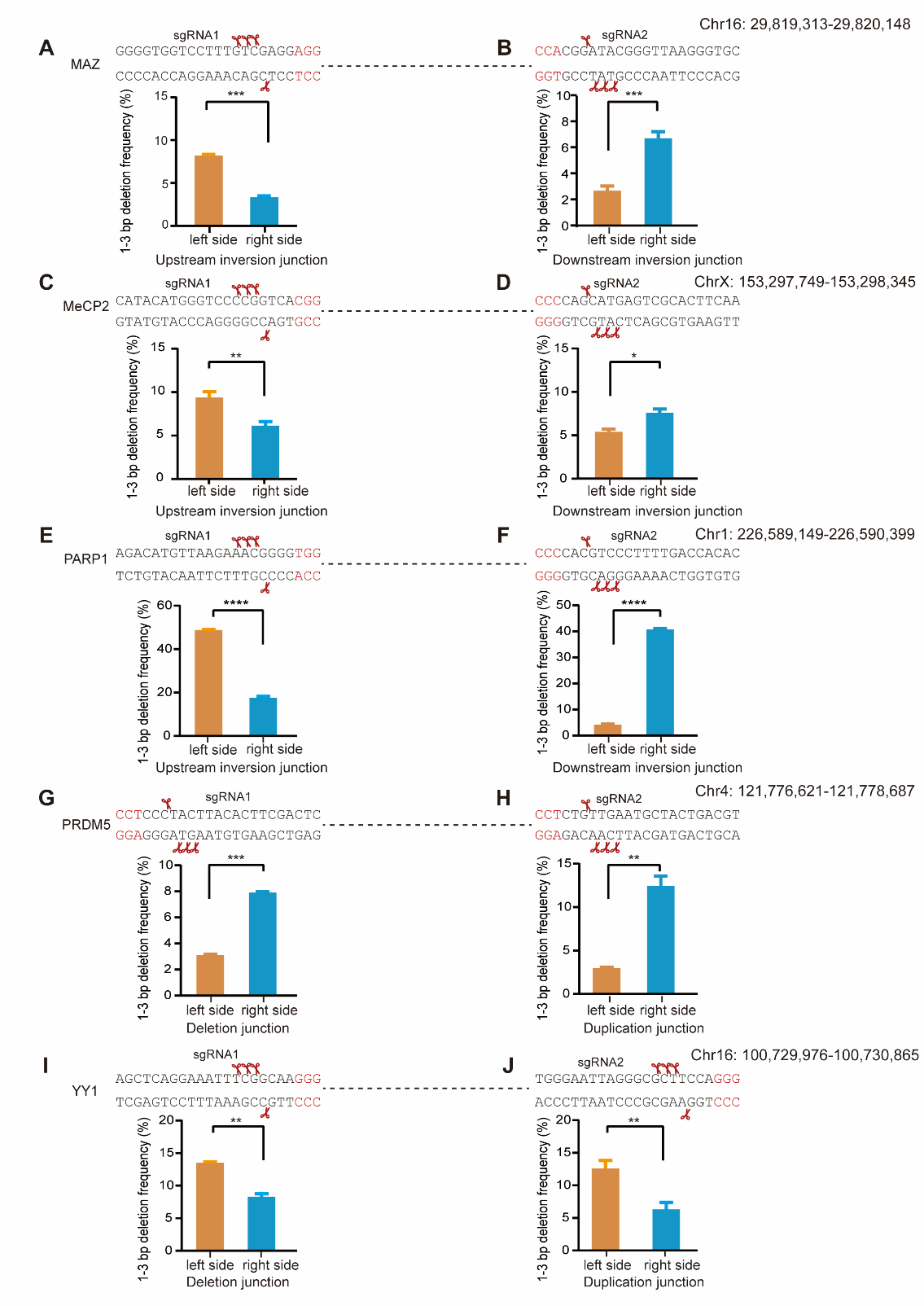


**Fig. S10** Biased deletion of nucleotides at junctional sites of chromosomal rearrangements confirms the staggered Cas9 cleavages. 1-3bp deletion frequencies at the chromosomal rearrangement junctions are always biased toward -4, -5, and -6 positions upstream of the PAM site of both sgRNAs at the *MAZ* (**A**, **B**), *MeCP2* (**C**, **D**), *PARP1* (**E**, **F**), *PRDM5* (**G**, **H**) and *YY1* (**I**, **J**) loci (see Additional file 5: Table S3, n = 3 replicates, mean ± SEM).


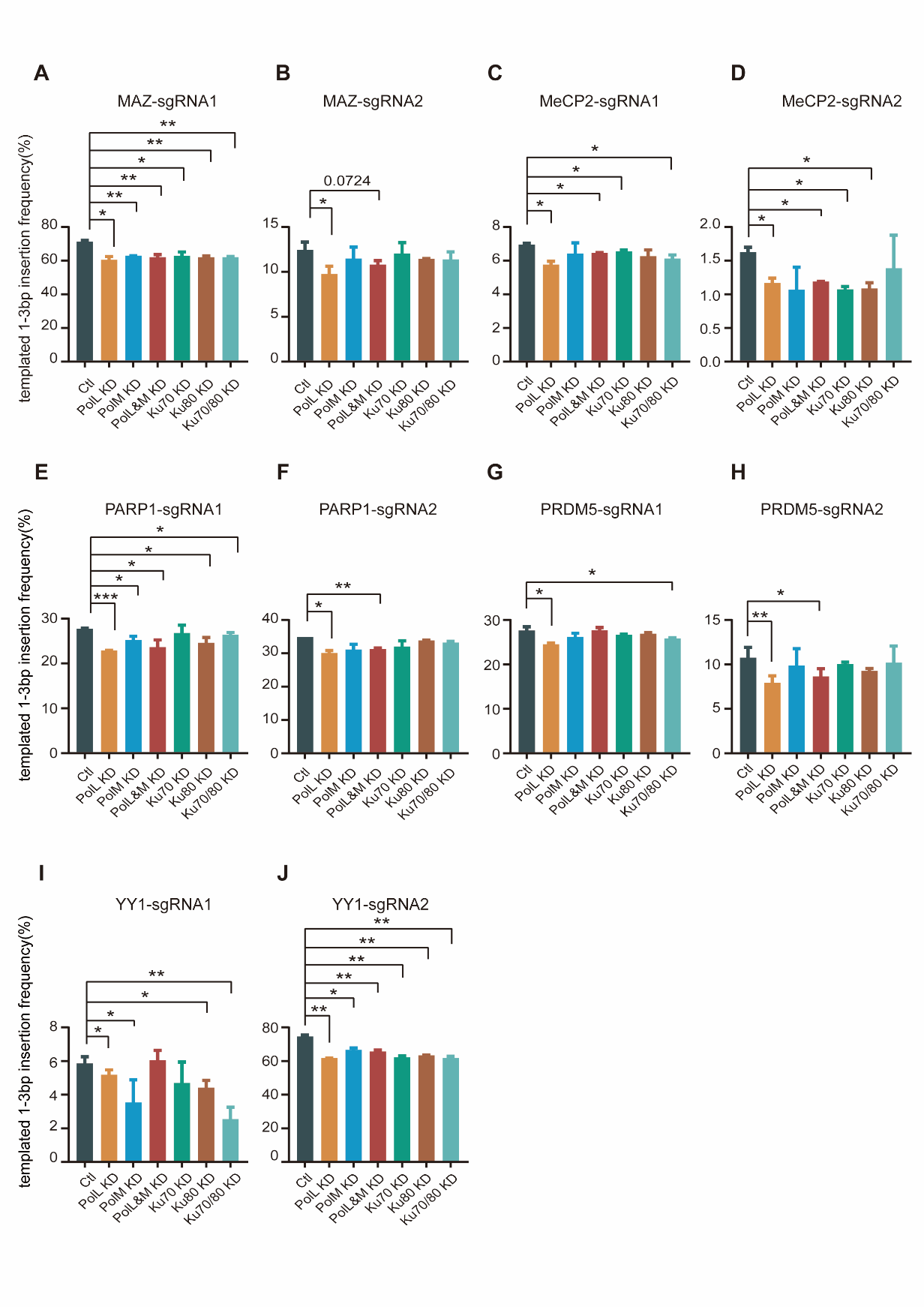


**Fig. S11** Fill-in of staggered Cas9 DSB ends by Polλ in HEK293T cells. Significant decreases of templated 1-3bp insertions at Cas9 cleavage junctions programmed with dual sgRNAs at the *MAZ* (**A**, **B**), *MeCP2* (**C**, **D**)*, PARP1* (**E**, **F**), *PRDM5* (**G**, **H**), and *YY1* (**I**, **J**) loci upon *PolL* knockdown (see Additional file 5: Table S3, n = 2 replicates, mean ± SEM). As positive controls, the trends of templated insertions upon Ku70/80-knockdown are similar as those of Polλ-knockdown.


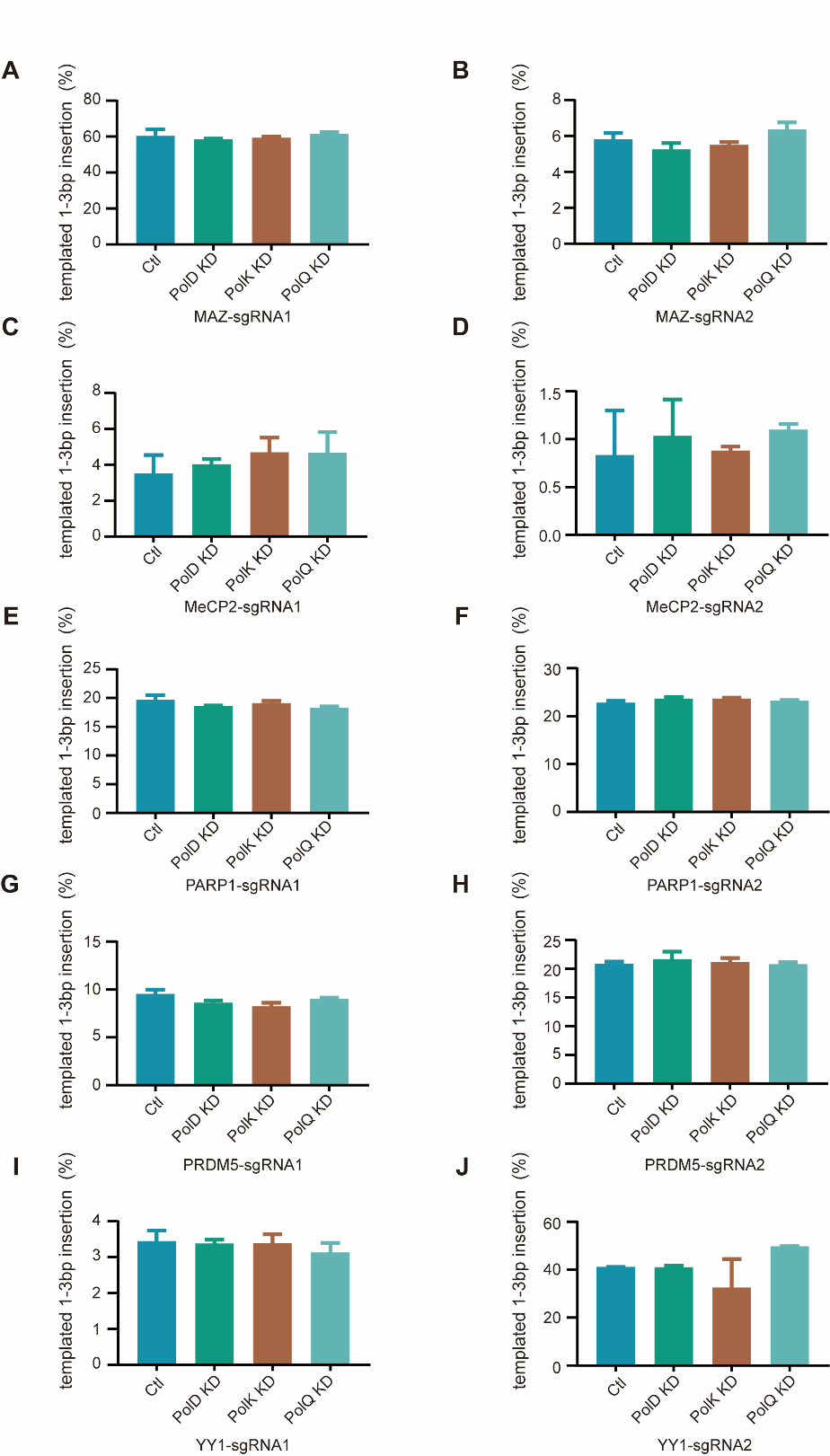


**Fig. S12** Polδ, Polκ, and Polθ are not engaged in the fill-in of staggered Cas9 cleavage ends. There is no significant difference of 1-3bp templated insertions at the junctional sites of chromosomal rearrangements programmed with Cas9 and dual sgRNAs at the *MAZ* (**A**, **B**), *MeCP2* (**C**, **D**), *PARP1* (**E**, **F**), *PRDM5* (**G**, **H**), and *YY1* (**I**, **J**) loci upon knockdown of the *PolD* (encoding Polδ polymerase), *PolK* (encoding Polκ polymerase), or *PolQ* (encoding Polθ polymerase) gene (see Additional file 5: Table S3, n = 3 replicates, mean ± SEM).


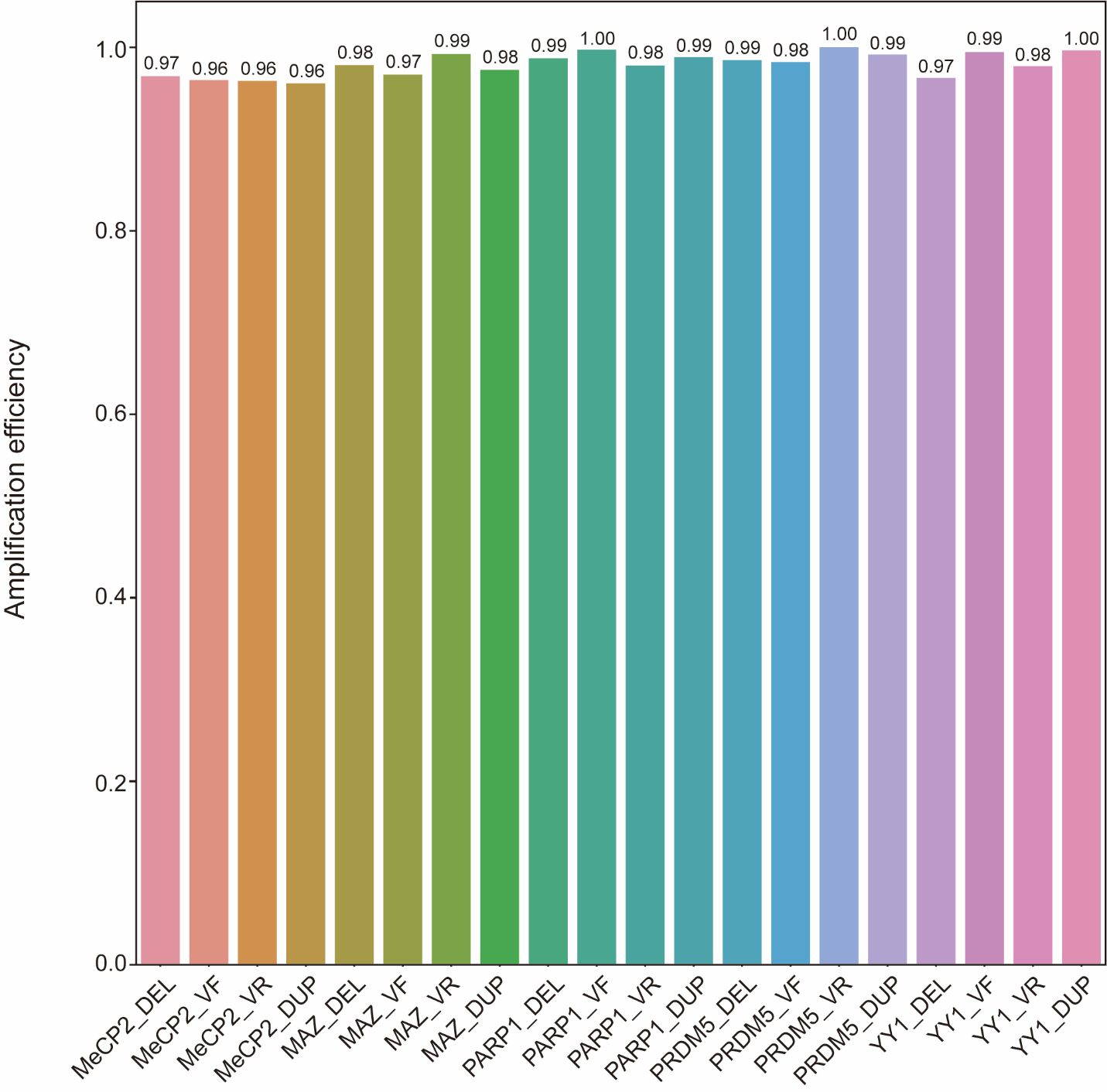


**Fig. S13** PCR modelling-based analysis by the pcrEfficiency software. The results showed that there is no apparent PCR amplification bias.
