# Supplementary material for: DNA polymerases in precise and predictable CRISPR/Cas9-mediated chromosomal rearrangements
Source: BMC Biol. 2023 Dec 8;21:288. doi: 10.1186/s12915-023-01784-y (PMC10709867; doi:10.1186/s12915-023-01784-y)
Supplement: Supplementary file 3 — Additional file 3: Table S1. Oligonucleotide sequences used in this study. [file 12915_2023_1784_MOESM3_ESM.docx]

**Table S1.** Oligonucleotide sequences used in this study.

|  | |
| --- | --- |
| Oligos for knocking down polymerases | |
| PL-E5-F | ACCGAGGCTCACAATCTCCCTCAA |
| PL-E5-R | AAACTTGAGGGAGATTGTGAGCCT |
| PL-E7-F | ACCGCTTCCTGGAACGTATGCCCA |
| PL-E7-R | AAACTGGGCATACGTTCCAGGAAG |
| PM-E10-F | ACCGGCTGCGCCGCTTCAGCCGGA |
| PM-E10-R | AAACTCCGGCTGAAGCGGCGCAGC |
| PM-E7-F | ACCGTACATCGGACCGCAGGACTG |
| PM-E7-R | AAACCAGTCCTGCGGTCCGATGTA |
| PQ-E16-F | ACCGACGCTCCAGAGTCTTTCAGG |
| PQ-E16-R | AAACCCTGAAAGACTCTGGAGCGT |
| PQ-E15-F | ACCGTTAGTAAGGATACCCGAACC |
| PQ-E15-R | AAACGGTTCGGGTATCCTTACTAA |
| PD1-E20-1F | ACCGGTATGGGAAGTAGACCTGGG |
| PD1-E20-1R | AAACCCCAGGTCTACTTCCCATAC |
| PD1-E22-1F | ACCGTGATGATCACGTAGGGGACG |
| PD1-E22-1R | AAACCGTCCCCTACGTGATCATCA |
| POLK-E2-F | ACCGGATGATCTTCTGCTTAGGAT |
| POLK-E2-R | AAACATCCTAAGCAGAAGATCATC |
| POLK-E3-F | ACCGATTGTGCTTTTCTTAGCTGT |
| POLK-E3-R | AAACACAGCTAAGAAAAGCACAAT |
| XRCC5-sg2-F | ACCGCATGGCCACCTAAGCGAAAG |
| XRCC5-sg2-R | aaacCTTTCGCTTAGGTGGCCATG |
| XRCC5-sg4-F | accgCCAGACTGGAGACGCTGAAG |
| XRCC5-sg4-R | aaacCTTCAGCGTCTCCAGTCTGG |
| XRCC6-sg2-F | accgGTTGCGGAAGGTTCGCGCCA |
| XRCC6-sg2-R | aaacTGGCGCGAACCTTCCGCAAC |
| XRCC6-sg4-F | accgAGCAGTGGACCTGACATGTA |
| XRCC6-sg4-R | aaacTACATGTCAGGTCCACTGCT |
| RT-PCR primers | |
| HPRT1-F | TGCTGACCTGCTGGATTACA |
| HPRT1-R | ACAATCCGCCCAAAGGGAAC |
| DCK-F | AGAGAAGCTGCCCGTCTTTC |
| DCK-R | CAGGAGCCAGCTTTCATGTT |
| Q-POLK-F | GCAGGCATTGCCCCAAATAC |
| Q-POLK-R | AAGCAATGCCCTCTGTTGGT |
| Q-POLL-F | GTCTGTGTTGGAGCAGCC |
| Q-POLL-R | CTTCTCAAAGAGTTCTGCCCG |
| Q-POLD-F | CGCAGTACTACCTGGAGCAG |
| Q-POLD-R | CGCATGTAGAAGATGGGGCA |
| Q-POLQ-F2 | TCCAGATTCTCAGGAGCCGA |
| Q-POLQ-R2 | GGGCATTTCCACCTTACGGA |
| Q-POLM-F2 | GCATTTTCCGCCTACCACAAC |
| Q-POLM-R2 | GGGTGCAACTACCAAGTCCAC |
| Q-XRCC5-F1 | GCTGACTTCCTGGATGCACT |
| Q-XRCC5-R1 | CTAAGCGAAAGGGGCCATCT |
| Q-XRCC6-F1 | CCAACATGTCAGGGTGGGAG |
| Q-XRCC6-R1 | ATAGCCTTGGAGGCATCAACC |
| GAPDH-F | GGAGTCCACTGGCGTCTTCAC |
| GAPDH-R | GCAGGAGGCATTGCTGATGAT |
| sgRNA sequences for the induction of chromosomal rearrangements | |
| MeCP2-sgRNA1-F | ACCGCATACATGGGTCCCCGGTCA |
| MeCP2-sgRNA1-R | AAACTGACCGGGGACCCATGTATG |
| MeCp2-sgRNA2-F | ACCGTTGAAGTGCGACTCATGCTG |
| MeCP2-sgRNA2-R | AAACCAGCATGAGTCGCACTTCAA |
| MAZ-sgRNA1-F | ACCGGGGGTGGTCCTTTGTCGAGG |
| MAZ-sgRNA1-R | AAACGGGGTGGTCCTTTGTCGAGG |
| MAZ-sgRNA2-F | ACCGGCACCCTTAACCCGTATCCG |
| MAZ-sgRNA2-R | AAACCGGATACGGGTTAAGGGTGC |
| PARP1-sgRNA1-F | ACCGAGACATGTTAAGAAACGGGG |
| PARP1-sgRNA1-R | AAACCCCCGTTTCTTAACATGTCT |
| PARP1-sgRNA2-F | ACCGGTGTGGTCAAAAGGGACGTG |
| PARP1-sgRNA2-R | AAACCACGTCCCTTTTGACCACAC |
| PRDM5-sgRNA1-F | ACCGGAGTCGAAGTGTAAGTAGGG |
| PRDM5-sgRNA1-R | AAACCCCTACTTACACTTCGACTC |
| PRDM5-sgRNA2-F | ACCGACGTCAGTAGCATTCAACAG |
| PRDM5-sgRNA2-R | AAACCTGTTGAATGCTACTGACGT |
| YY1-sgRNA1-F | ACCGAGCTCAGGAAATTTCGGCAA |
| YY1-sgRNA1-R | AAACTTGCCGAAATTTCCTGAGCT |
| YY1-sgRNA2-F | ACCGTGGGAATTAGGGCGCTTCCA |
| YY1-sgRNA2-R | AAACTGGAAGCGCCCTAATTCCCA |
| Oligos for the construction libraries for high throughput sequencing | |
| Hiseq-MECP2-P7F1 | CAAGCAGAAGACGGCATACGAGATCGTACAGTGACTGGAGTTCAGACGTGTGCTCTTCCGATCTCTGCCCTGTAGAGATAGGAGTTG |
| Hiseq-MECP2-P7F1-a | CAAGCAGAAGACGGCATACGAGATGATCTGGTGACTGGAGTTCAGACGTGTGCTCTTCCGATCTCTGCCCTGTAGAGATAGGAGTTG |
| Hiseq-MECP2-P7R1 | CAAGCAGAAGACGGCATACGAGATGTAGCTGTGACTGGAGTTCAGACGTGTGCTCTTCCGATCTGCAAAGCAGAGACATCAGAAGG |
| Hiseq-MECP2-P7R1-a | CAAGCAGAAGACGGCATACGAGATCTTGTAGTGACTGGAGTTCAGACGTGTGCTCTTCCGATCTGCAAAGCAGAGACATCAGAAGG |
| HTSD- MECP2-P5F1 | AATGATACGGCGACCACCGAGATCTACACGACCTGTAACACTCTTTCCCTACACGACGCTCTTCCGATCTTCCTCTGAGCGGAAACACTG |
| HTSD- MECP2-P5F1-a | AATGATACGGCGACCACCGAGATCTACACATGTAACTACACTCTTTCCCTACACGACGCTCTTCCGATCTCGTCCTCTGAGCGGAAACACTG |
| HTSD- MECP2-P5F1-b | AATGATACGGCGACCACCGAGATCTACACGTTTCAGAACACTCTTTCCCTACACGACGCTCTTCCGATCTAGTTCCTCTGAGCGGAAACACTG |
| HTSD- MECP2-P5F1-c | AATGATACGGCGACCACCGAGATCTACACCACAGGATACACTCTTTCCCTACACGACGCTCTTCCGATCTTACGTCCTCTGAGCGGAAACACTG |
| HTSD-MECP2-P5R1 | AATGATACGGCGACCACCGAGATCTACACTAGCTGCCACACTCTTTCCCTACACGACGCTCTTCCGATCTCTCTTGTCAGCCATCGAGCC |
| HTSD-MECP2-P5R1-a | AATGATACGGCGACCACCGAGATCTACACAGCGAATGACACTCTTTCCCTACACGACGCTCTTCCGATCTCTCTCTTGTCAGCCATCGAGCC |
| HTSD-MECP2-P5R1-b | AATGATACGGCGACCACCGAGATCTACACTATGCTGCACACTCTTTCCCTACACGACGCTCTTCCGATCTACACTCTTGTCAGCCATCGAGCC |
| HTSD-MECP2-P5R1-c | AATGATACGGCGACCACCGAGATCTACACAGAAGACTACACTCTTTCCCTACACGACGCTCTTCCGATCTTCGACTCTTGTCAGCCATCGAGCC |
| Hiseq-MAZ-P7F1 | CAAGCAGAAGACGGCATACGAGATGTCGAAGTGACTGGAGTTCAGACGTGTGCTCTTCCGATCTACTGTGGCAAGAGCTTCTCC |
| Hiseq-MAZ-P7F1-a | CAAGCAGAAGACGGCATACGAGATAGGAATGTGACTGGAGTTCAGACGTGTGCTCTTCCGATCTACTGTGGCAAGAGCTTCTCC |
| Hiseq-MAZ-P7R1 | CAAGCAGAAGACGGCATACGAGATGCAATCGTGACTGGAGTTCAGACGTGTGCTCTTCCGATCTCTTAAGCAGGAAATCCCTCCCC |
| Hiseq-MAZ-P7R1-a | CAAGCAGAAGACGGCATACGAGATACTTGAGTGACTGGAGTTCAGACGTGTGCTCTTCCGATCTCTTAAGCAGGAAATCCCTCCCC |
| HTSD-MAZ-P5F1 | AATGATACGGCGACCACCGAGATCTACACGACCTGTAACACTCTTTCCCTACACGACGCTCTTCCGATCTACAAAGGTACATGCCGAGGG |
| HTSD-MAZ-P5F1-a | AATGATACGGCGACCACCGAGATCTACACATGTAACTACACTCTTTCCCTACACGACGCTCTTCCGATCTTAACAAAGGTACATGCCGAGGG |
| HTSD-MAZ-P5F1-b | AATGATACGGCGACCACCGAGATCTACACGTTTCAGAACACTCTTTCCCTACACGACGCTCTTCCGATCTCCGACAAAGGTACATGCCGAGGG |
| HTSD-MAZ-P5F1-c | AATGATACGGCGACCACCGAGATCTACACCACAGGATACACTCTTTCCCTACACGACGCTCTTCCGATCTGATCACAAAGGTACATGCCGAGGG |
| HTSD-MAZ-P5R1 | AATGATACGGCGACCACCGAGATCTACACTAGCTGCCACACTCTTTCCCTACACGACGCTCTTCCGATCTTCCCTTGACCTCTTGTAGGAATCT |
| HTSD-MAZ-P5R1-a | AATGATACGGCGACCACCGAGATCTACACAGCGAATGACACTCTTTCCCTACACGACGCTCTTCCGATCTGATCCCTTGACCTCTTGTAGGAATCT |
| HTSD-MAZ-P5R1-b | AATGATACGGCGACCACCGAGATCTACACTATGCTGCACACTCTTTCCCTACACGACGCTCTTCCGATCTCACTCCCTTGACCTCTTGTAGGAATCT |
| HTSD-MAZ-P5R1-c | AATGATACGGCGACCACCGAGATCTACACAGAAGACTACACTCTTTCCCTACACGACGCTCTTCCGATCTATGCTCCCTTGACCTCTTGTAGGAATCT |
| Hiseq-PARP1-P7F1 | CAAGCAGAAGACGGCATACGAGATTCAGGTGTGACTGGAGTTCAGACGTGTGCTCTTCCGATCTGCTTTATTGAGGCAGCAGTGTTATG |
| Hiseq-PARP1-P7F1-a | CAAGCAGAAGACGGCATACGAGATCACTGTGTGACTGGAGTTCAGACGTGTGCTCTTCCGATCTGCTTTATTGAGGCAGCAGTGTTATG |
| Hiseq-PARP1-P7R1 | CAAGCAGAAGACGGCATACGAGATGGAACTGTGACTGGAGTTCAGACGTGTGCTCTTCCGATCTACTAACTAAAGCAGGGACAGGG |
| Hiseq-PARP1-P7R1-a | CAAGCAGAAGACGGCATACGAGATATGAGCGTGACTGGAGTTCAGACGTGTGCTCTTCCGATCTACTAACTAAAGCAGGGACAGGG |
| HTSD-PARP1-P5F1 | AATGATACGGCGACCACCGAGATCTACACGACCTGTAACACTCTTTCCCTACACGACGCTCTTCCGATCTTCTCTTAGGACACCAAACACAGC |
| HTSD-PARP1-P5F1-a | AATGATACGGCGACCACCGAGATCTACACATGTAACTACACTCTTTCCCTACACGACGCTCTTCCGATCTACTCTCTTAGGACACCAAACACAGC |
| HTSD-PARP1-P5F1-b | AATGATACGGCGACCACCGAGATCTACACGTTTCAGAACACTCTTTCCCTACACGACGCTCTTCCGATCTGGCTCTCTTAGGACACCAAACACAGC |
| HTSD-PARP1-P5F1-c | AATGATACGGCGACCACCGAGATCTACACCACAGGATACACTCTTTCCCTACACGACGCTCTTCCGATCTTCTGTCTCTTAGGACACCAAACACAGC |
| HTSD-PARP1-P5R1 | AATGATACGGCGACCACCGAGATCTACACTAGCTGCCACACTCTTTCCCTACACGACGCTCTTCCGATCTCATGAGGCAGCTCACCACTAA |
| HTSD-PARP1-P5R1-a | AATGATACGGCGACCACCGAGATCTACACAGCGAATGACACTCTTTCCCTACACGACGCTCTTCCGATCTGCCATGAGGCAGCTCACCACTAA |
| HTSD-PARP1-P5R1-b | AATGATACGGCGACCACCGAGATCTACACTATGCTGCACACTCTTTCCCTACACGACGCTCTTCCGATCTAAGCATGAGGCAGCTCACCACTAA |
| HTSD-PARP1-P5R1-c | AATGATACGGCGACCACCGAGATCTACACAGAAGACTACACTCTTTCCCTACACGACGCTCTTCCGATCTCTTGCATGAGGCAGCTCACCACTAA |
| Hiseq-PRDM5-P7F | CAAGCAGAAGACGGCATACGAGATTGACCAGTGACTGGAGTTCAGACGTGTGCTCTTCCGATCTCCATCACTGGGAAGCACGAA |
| Hiseq-PRDM5-P7F-a | CAAGCAGAAGACGGCATACGAGATACAGTGGTGACTGGAGTTCAGACGTGTGCTCTTCCGATCTCCATCACTGGGAAGCACGAA |
| Hiseq-PRDM5-P7R | CAAGCAGAAGACGGCATACGAGATTGCCATGTGACTGGAGTTCAGACGTGTGCTCTTCCGATCTTTACCATATCAGTGTTGCTGGACA |
| Hiseq-PRDM5-P7R-a | CAAGCAGAAGACGGCATACGAGATAAGCTAGTGACTGGAGTTCAGACGTGTGCTCTTCCGATCTTTACCATATCAGTGTTGCTGGACA |
| HTSD-PRDM5-P5F3 | AATGATACGGCGACCACCGAGATCTACACGACCTGTAACACTCTTTCCCTACACGACGCTCTTCCGATCTTTCATGTCTGTATGACTTTGCTGC |
| HTSD-PRDM5-P5F3-a | AATGATACGGCGACCACCGAGATCTACACATGTAACTACACTCTTTCCCTACACGACGCTCTTCCGATCTTATTCATGTCTGTATGACTTTGCTGC |
| HTSD-PRDM5-P5F3-b | AATGATACGGCGACCACCGAGATCTACACGTTTCAGAACACTCTTTCCCTACACGACGCTCTTCCGATCTGCATTCATGTCTGTATGACTTTGCTGC |
| HTSD-PRDM5-P5F3-c | AATGATACGGCGACCACCGAGATCTACACCACAGGATACACTCTTTCCCTACACGACGCTCTTCCGATCTCGGTTTCATGTCTGTATGACTTTGCTGC |
| HTSD-PRDM5-P5R3 | AATGATACGGCGACCACCGAGATCTACACTAGCTGCCACACTCTTTCCCTACACGACGCTCTTCCGATCTTGACCAGCACATTATTTCTCTCAGA |
| HTSD-PRDM5-P5R3-a | AATGATACGGCGACCACCGAGATCTACACAGCGAATGACACTCTTTCCCTACACGACGCTCTTCCGATCTGTTGACCAGCACATTATTTCTCTCAGA |
| HTSD-PRDM5-P5R3-b | AATGATACGGCGACCACCGAGATCTACACTATGCTGCACACTCTTTCCCTACACGACGCTCTTCCGATCTATATGACCAGCACATTATTTCTCTCAGA |
| HTSD-PRDM5-P5R3-c | AATGATACGGCGACCACCGAGATCTACACAGAAGACTACACTCTTTCCCTACACGACGCTCTTCCGATCTTGAGTGACCAGCACATTATTTCTCTCAGA |
| HTSD-YY1-P5F1 | AATGATACGGCGACCACCGAGATCTACACGACCTGTAACACTCTTTCCCTACACGACGCTCTTCCGATCTAACTCTGGAGCAGAAAGCCTAAT |
| HTSD-YY1-P5F1-a | AATGATACGGCGACCACCGAGATCTACACATGTAACTACACTCTTTCCCTACACGACGCTCTTCCGATCTTGAACTCTGGAGCAGAAAGCCTAAT |
| HTSD-YY1-P5F1-b | AATGATACGGCGACCACCGAGATCTACACGTTTCAGAACACTCTTTCCCTACACGACGCTCTTCCGATCTGCTAACTCTGGAGCAGAAAGCCTAAT |
| HTSD-YY1-P5F1-c | AATGATACGGCGACCACCGAGATCTACACCACAGGATACACTCTTTCCCTACACGACGCTCTTCCGATCTAACTAACTCTGGAGCAGAAAGCCTAAT |
| HTSD-YY1-P5R1 | AATGATACGGCGACCACCGAGATCTACACTAGCTGCCACACTCTTTCCCTACACGACGCTCTTCCGATCTGCAACCCACTCTGTTCTTAGAATG |
| HTSD-YY1-P5R1-a | AATGATACGGCGACCACCGAGATCTACACAGCGAATGACACTCTTTCCCTACACGACGCTCTTCCGATCTATGCAACCCACTCTGTTCTTAGAATG |
| HTSD-YY1-P5R1-b | AATGATACGGCGACCACCGAGATCTACACTATGCTGCACACTCTTTCCCTACACGACGCTCTTCCGATCTCGTGCAACCCACTCTGTTCTTAGAATG |
| HTSD-YY1-P5R1-c | AATGATACGGCGACCACCGAGATCTACACAGAAGACTACACTCTTTCCCTACACGACGCTCTTCCGATCTTGCAGCAACCCACTCTGTTCTTAGAATG |
| Hiseq-YY1-P7F2 | CAAGCAGAAGACGGCATACGAGATCGATGTGTGACTGGAGTTCAGACGTGTGCTCTTCCGATCTCTCCTGACCTAAAGTGATCCACC |
| Hiseq-YY1-P7F2-a | CAAGCAGAAGACGGCATACGAGATCGAAACGTGACTGGAGTTCAGACGTGTGCTCTTCCGATCTCTCCTGACCTAAAGTGATCCACC |
| Hiseq-YY1-P7R2 | CAAGCAGAAGACGGCATACGAGATTTAGGCGTGACTGGAGTTCAGACGTGTGCTCTTCCGATCTCTCTACTCACACAGCCCCTTC |
| Hiseq-YY1-P7R2-a | CAAGCAGAAGACGGCATACGAGATCAGATCGTGACTGGAGTTCAGACGTGTGCTCTTCCGATCTCTCTACTCACACAGCCCCTTC |
| Primers for linear amplifications followed by high throughput genome wide translocation sequencing | |
| Nested-HPRT1-E2F-a | AATGATACGGCGACCACCGAGATCTACACTCTTTCCCTACACGACGCTCTTCCGATCTGCTACCATGCTGAGGATTTGGAAAGGG |
| Nested-HPRT1-E2F-b | AATGATACGGCGACCACCGAGATCTACACTCTTTCCCTACACGACGCTCTTCCGATCTGCTGCACTGCTGAGGATTTGGAAAGGGT |
| Nested-HPRT1-E2F-c | AATGATACGGCGACCACCGAGATCTACACTCTTTCCCTACACGACGCTCTTCCGATCTGCTATCGTGCTGAGGATTTGGAAAGGGT |
| Nested-HPRT1-E2F-d | AATGATACGGCGACCACCGAGATCTACACTCTTTCCCTACACGACGCTCTTCCGATCTGATTCATTGCTGAGGATTTGGAAAGGGT |
| P7-A | CAAGCAGAAGACGGCATACGAGATTCGTACGTGACTGGAGTTCAGACGTGTGCTCTTCCGATCT |
| P7-B | CAAGCAGAAGACGGCATACGAGATTCAGACGTGACTGGAGTTCAGACGTGTGCTCTTCCGATCT |
| P7-C | CAAGCAGAAGACGGCATACGAGATTCGCATGTGACTGGAGTTCAGACGTGTGCTCTTCCGATCT |
| P7-D | CAAGCAGAAGACGGCATACGAGATCGAGAGGTGACTGGAGTTCAGACGTGTGCTCTTCCGATCT |
| Adapter-upper | GACGTGTGCTCTTCCGATCTGNNNNNN |
| Adapter-lower | CAGATCGGAAGAGCACACGTC |
| Bio-HPRT-E2F | TGTTTGTATCCTGTAATGCTCTCA |
| sgRNA sequences for cell growth assay | |
| HPRT1-E2-sgRNA-F | ACCGTTGCTATTTGAACATAAACT |
| HPRT1-E2-sgRNA-R | AAACAGTTTATGTTCAAATAGCAA |
| HPRT1-E3-sgRNA-F | ACCGGTGGAAGTTTAATGACTAAG |
| HPRT1-E3-sgRNA-R | AAACCTTAGTCATTAAACTTCCAC |
| HPRT1-E3-in-F | ACCGGCCCCCCTTGAGCACACAGA |
| HPRT1-E3-in-R | AAACTCTGTGTGCTCAAGGGGGGC |
| HPRT1-D-E2-F | ACCGAGTATCAGTTGTGGTATAGT |
| HPRT1-D-E2-R | AAACACTATACCACAACTGATACT |
| DCK-E4-D-sgRNA-F | ACCGATATTTAGAACTCTTTTCAG |
| DCK-E4-D-sgRNA-R | AAACCTGAAAAGAGTTCTAAATAT |
| DCK-E5-U-sgRNA-F | ACCGAGAGATGGAAGAAAAAGGCA |
| DCK-E5-U-sgRNA-R | AAACTGCCTTTTTCTTCCATCTCT |
| DCK-E4-in-F | ACCGAGTGGACAATTTATCAAGAC |
| DCK-E4-in-R | AAACGTCTTGATAAATTGTCCACT |
| SgRNA sequences for the induction of chromosomal rearrangements for linear amplifications | |
| HPRT1-sgRNA1-F | ACCGGTAAGTAAGATCTTAAAATG |
| HPRT1-sgRNA1-R | AAACCATTTTAAGATCTTACTTAC |
| HPRT1-sgRNA2-F | ACCGAGTCCTACAGAAATAAAATC |
| HPRT1-sgRNA2-R | AAACGATTTTATTTCTGTAGGACT |
